# Personality traits measured by the HEXACO personality inventory and the dark triad predict university students’ attitudes and misconduct behaviors related to generative artificial intelligence: Haiying Liang^1^, Xu Mao^2^, Michael J. Reiss^3*^

**DOI:** 10.1038/s41598-025-25744-4

**Published:** 2025-11-25

**Authors:** Haiying Liang, Xu Mao, Michael J. Reiss

**Affiliations:** 1 School of Foreign Languages, Peking University, Beijing, China; 2 School of Health Humanities, Peking University, Beijing, China; 3 Institute of Education, University College London, London, UK

**Keywords:** Psychology, Psychology, Science, technology and society

## Abstract

This study investigates how personality traits, specifically those measured by the HEXACO Personality Inventory and the Dark Triad, predict university students’ attitudes toward generative artificial intelligence (GAI) and their engagement in GAI-related academic misconduct. The first objective was to develop and validate a Chinese-language scale to measure students’ attitudes toward GAI in academic contexts. The newly developed GAI Attitudes Scale was tested for psychometric properties, showing high internal consistency (Cronbach’s α = 0.92) and reliability. In the second part of the study, hierarchical linear regression analyses explored the relationship between personality traits and both GAI attitudes and misconduct behaviors. Findings indicated that Extraversion and Openness to Experience were significant positive predictors of favorable GAI attitudes. Regarding misconduct behaviors, Honesty-Humility, Agreeableness, and Conscientiousness were significant negative predictors, while Narcissism and Psychopathy were significant positive predictors. Notably, GAI attitudes did not provide additional predictive value for misconduct beyond personality traits. Taken together, the findings demonstrate that personality traits are central to understanding both the adoption and misuse of GAI in academic contexts, providing important insights for fostering ethical engagement with emerging technologies.

**Supplementary Information:**

The online version contains supplementary material available at 10.1038/s41598-025-25744-4.

## Introduction

The rapid proliferation of generative artificial intelligence (GAI) technologies is fundamentally transforming the way people engage with information. As a specialized subfield of artificial intelligence (AI), GAI is distinguished by its capacity to autonomously generate coherent and contextually relevant content^1^. As GAI becomes increasingly integrated into students’ academic practices—including literature reviews, manuscript drafting, code generation, and automated feedback^2^—it is vital to understand how they perceive and use these tools. Such inquiry is essential not only for educators and policymakers but also for guiding the ethical integration of GAI into scholarly practices. However, rigorous investigation into these attitudes depends on the availability of psychometrically robust, theoretically grounded instruments specifically tailored to GAI. While several instruments have been developed to assess general attitudes toward AI—such as the General Attitudes Toward Artificial Intelligence Scale^3^, the Attitudes Toward Artificial Intelligence Scale^4^, and the more recent Attitudes Towards AI scale-12 by Stein et al.^5^—these tools typically treat AI as a broad and undifferentiated construct, without differentiating the specific characteristics of GAI. Furthermore, few of these instruments were developed within academic contexts, and most do not capture the tripartite structure of attitude (cognitive, affective, behavioral) foundational in social psychology^6^. Additionally, existing measures are almost exclusively developed in English, limiting their applicability across linguistic and cultural boundaries.

To address these gaps, the first objective of this study was to develop and validate the GAI Attitudes Scale—a concise Chinese-language instrument that assesses students’ attitudes toward GAI specifically within academic contexts. The scale is designed to capture both positive and negative evaluations of GAI, grounded in the tripartite model of attitude, and to conceptualize GAI as a general set of technological affordances rather than as specific platforms (e.g., ChatGPT or Midjourney). This abstraction allows for broader applicability across disciplines and reduces the influence of transient technological branding on participants’ evaluations.

Accordingly, the first research objective was: to construct and validate a psychometric instrument that captures students’ attitudes toward GAI within academic settings.

While prior research on technology adoption has largely focused on demographic and sociocultural factors—such as age, gender, education level, and media exposure^7–10^—psychological dispositions—particularly personality traits—have received comparatively less attention in this domain. Nevertheless, a growing body of evidence has shown that certain personality dimensions (e.g., openness, conscientiousness, extraversion) are associated with users’ technology-related attitudes and behaviors^11,12^. However, research specifically examining how broad personality frameworks such as HEXACO and the Dark Triad predict attitudes and ethical behaviors toward generative AI tools remains limited. Addressing this gap, the present study investigates how these personality traits shape students’ evaluative and behavioral responses to GAI.

Previous research has primarily relied on the Big Five personality model to examine technology acceptance^5,13–15^. While the Big Five model offers valuable insights into broad dispositional tendencies, it may fall short in capturing morally relevant personality dimensions when measuring attitudes toward AI. As Stein et al.^5^ suggest, the HEXACO Personality Inventory may provide more nuanced explanatory power, particularly through its inclusion of the Honesty-Humility dimension—a trait strongly linked to ethical decision-making and adherence to rules.

While the Dark Triad traits (Machiavellianism, Psychopathy and Narcissism) have been applied in studies of technology abuse^16–18^, their use in the context of GAI-related misconduct remains limited. In academic settings, these three traits may manifest in distinctive ways when students engage with GAI. For instance, a Machiavellian student, driven by strategic manipulation and self-interest^19^, might exploit GAI tools to generate essays or complete assignments with the primary aim of securing higher grades, while concealing their misconduct from instructors. A narcissistic student, characterized by grandiosity and a need for admiration^19^, might justify or even boast about using GAI, framing it as evidence of their “superior” intelligence and innovative capabilities, regardless of ethical concerns. In contrast, a student high in psychopathy, marked by impulsivity, callousness, and a lack of remorse^19^, might disregard academic integrity altogether, using GAI to plagiarize or fabricate work without guilt or consideration of potential consequences. Together, these traits highlight the socially aversive motivations that can drive academic misconduct in the context of emerging technologies such as GAI.

Accordingly, the second objective of this study was: to examine the predictive roles of personality—assessed via the HEXACO Personality Inventory and the Dark Triad traits—in shaping students’ attitudes toward GAI and their engagement in GAI-related misconduct.

This research seeks to identify which psychological profiles are more likely to embrace or misuse generative AI tools. Understanding these associations can contribute to the development of targeted interventions, ethical training, and evidence-based policy recommendations, improving the responsible use of GAI in education and scholarly communication.

## Overview of studies and theoretical predictions

### Overview of study 1

To address the two overarching research objectives, we conducted two empirical studies. Study 1 focuses on the development and validation of a novel instrument designed to measure students’ attitudes toward GAI in academic settings. Building upon and adapting the ATTARI-12 framework^5^, this study evaluated the psychometric properties of the new scale, including its internal consistency, test–retest reliability, and convergent validity. Additionally, we examined the potential influence of social desirability bias on self-reported GAI attitudes. These initial validation studies were critical for ensuring that the instrument accurately captures both the positive and negative dimensions of GAI evaluation within a research context.

### Overview of study 2

Study 2 extended this work by investigating how university students’ attitudes toward GAI and GAI misconduct behaviors relate to individual differences in personality measured through HEXACO and the Personality Inventory and the Dark Triad traits.

Accordingly, we formulated a series of hypotheses regarding how these personality dimensions may predict both attitudes towards GAI and engagement in GAI misconduct, as follows.

#### The HEXACO personality inventory

The HEXACO Personality Inventory is a widely used model in personality psychology, measuring six major traits: Honesty-Humility, Emotionality, Extraversion, Agreeableness, Conscientiousness, and Openness to Experience^20,21^. This model has shown strong reliability and validity across various cultural contexts and is useful for understanding human behaviors^22,23^.

*Honesty–Humility*, a core dimension of the HEXACO personality model, encompasses traits such as sincerity, fairness, and moral restraint. Individuals scoring high in this dimension are characterized by their reluctance to manipulate others for personal gain and their commitment to ethical behavior^23^. This trait has been shown to significantly influence ethical decision-making and pro-social behaviors^24^. In the context of GAI, researchers with high Honesty–Humility are expected to adopt more cautious attitudes toward GAI use. This expectation is grounded in evidence suggesting that individuals high in Honesty–Humility are less likely to engage in unethical behaviors^25^. Furthermore, these individuals are anticipated to hold more negative attitudes toward GAI misconduct behaviors. Therefore, it is hypothesized that:

##### H1

Students higher in Honesty–Humility will report more negative attitudes toward GAI.

##### H2

Students higher in Honesty–Humility will report lower likelihood of engaging in GAI misconduct behaviors.

*Emotionality* encompasses traits such as anxiety, fearfulness, dependence, and sentimentality^22,23^. Individuals with high Emotionality tend to experience heightened sensitivity to stress and a strong need for emotional support from others^26^. This heightened sensitivity may lead them to perceive greater risks and potential negative outcomes associated with GAI technologies. Consequently, they may exhibit more cautious attitudes toward GAI use, driven by concerns about potential misuse or unintended consequences. Research has shown that individuals high in Emotionality are more likely to avoid risky behaviors and seek reassurance in uncertain situations^27^. This tendency suggests that such individuals may be less inclined to engage in unethical practices involving GAI due to fears of detection and feelings of guilt. Therefore, it is hypothesized that:

##### H3

Students higher in Emotionality will report more negative attitudes toward GAI.

##### H4

Students higher in Emotionality will report lower likelihood of engaging in GAI misconduct behaviors.

*Extraversion*, characterized by sociability, enthusiasm, and assertiveness, is often associated with openness to innovation and experimentation^22,23^. Individuals high in extraversion are generally more willing to engage with new technologies, including GAI, due to their greater comfort in social interactions and openness to new experiences^5,28^. This inclination may lead them to adopt GAI more readily, especially in environments where such technologies are perceived as enhancing performance or are socially accepted. However, this same openness can also increase the likelihood of engaging in misconduct if the use of GAI is seen as socially tolerated or advantageous. Therefore, it is hypothesized that:

##### H5

Students higher in Extraversion will report more positive attitudes toward GAI.

##### H6

Students higher in Extraversion will report higher likelihood of engaging in GAI misconduct behaviors.

*Agreeableness* is characterized by traits such as empathy, cooperativeness, and trustworthiness^22,23^. Individuals high in agreeableness are more likely to engage in behaviors that promote social harmony and are less inclined to act unethically^16,29^. This tendency extends to their interactions with technology, where agreeable individuals may favor technologies that align with ethical standards and societal well-being. Research indicates that agreeableness is positively associated with prosocial behaviors and moral decision-making, suggesting that agreeable individuals are more likely to adopt technologies like GAI in ways that are ethically sound and socially responsible^26^. Furthermore, agreeableness has been linked to lower tendencies toward unethical behavior. Studies have shown that individuals high in agreeableness are less likely to engage in deviant behaviors, including those involving technology misuse^16,30^. This suggests that researchers with higher levels of agreeableness may be less inclined to misuse GAI and more likely to uphold ethical standards in their use of such technologies. Therefore, it is hypothesized that:

##### H7

Students higher in Agreeableness will report more positive attitudes toward GAI.

##### H8

Students higher in Agreeableness will report lower likelihood of engaging in GAI misconduct behaviors.

*Conscientiousness* is characterized by traits such as diligence, self-discipline, and ethical responsibility^22,23^. Highly conscientious individuals tend to be cautious and deliberate in their decision-making processes, often exhibiting skepticism toward technologies^16,31^. This skepticism arises from their preference for structured environments and adherence to established norms, leading them to critically assess the potential risks and implications of adopting new technologies^26^. Moreover, conscientious individuals are less likely to engage in misconduct related to GAI because their strong sense of duty and moral responsibility fosters adherence to ethical standards, reducing the likelihood of participating in activities such as academic dishonesty or misuse of AI-generated content^27^. Based on these considerations, the following hypotheses are proposed:

##### H9

Students higher in Conscientiousness will report more negative attitudes toward GAI.

##### H10

Students higher in Conscientiousness will report lower likelihood of engaging in GAI misconduct behaviors.

*Openness to Experience* is characterized by traits such as intellectual curiosity, creativity, and a preference for novelty^22,23^. Individuals high in Openness to Experience are typically more willing to explore and adopt innovative technologies^32^. This openness may lead to more positive attitudes toward GAI use, as these individuals are generally more accepting of technological advancements. However, Openness to Experience is not directly associated with unethical intent^33^. While individuals high in Openness to Experience tend to be intellectually curious and capable of evaluating the ethical risks of GAI misuse, this trait also encompasses a willingness to explore unconventional ideas and challenge existing norms. Hence, openness can operate in two directions. On the one hand, such individuals are more likely to critically evaluate the ethical risks and long-term societal consequences associated with GAI misuse^34^. Their broad thinking and reflective nature may lead them to adopt a more cautious and morally aware stance, resulting in more negative attitudes toward GAI misconduct. On the other hand, it may increase the likelihood of bending or breaking rules when individuals perceive GAI use as socially acceptable or instrumentally beneficial for academic success. The behavioral outcome thus depends largely on how the individual interprets prevailing social and academic norms surrounding GAI. Therefore, it is hypothesized that:

##### H11

Students higher in Openness to Experience will report more positive attitudes toward GAI.

##### H12

Students higher in Openness to Experience will not predict GAI misconduct behaviors.

#### The Dark Triad

The Dark Triad refers to a cluster of three interrelated but distinct personality traits—Machiavellianism, Psychopathy, and Narcissism—that are characterized by self-serving, manipulative, and often callous behaviors^35^. Coined by Paulhus and Williams^35^, the Dark Triad has become a central construct in personality psychology, particularly in understanding antisocial and socially aversive behaviors^35–37^.

*Machiavellianism* is characterized by manipulativeness, strategic self-interest, and a lack of morality^35^. Individuals exhibiting high levels of Machiavellianism tend to view interpersonal relationships as opportunities for exploitation, often employing deceitful tactics to achieve personal goals^38–40^. In the context of academic research, individuals high in Machiavellianism may be more inclined to exploit GAI technologies for competitive advantage, regardless of ethical considerations. Therefore, it is hypothesized that:

##### H13

Students higher in Machiavellianism will report more positive attitudes toward GAI.

##### H14

Students higher in Machiavellianism will report a higher likelihood of engaging in GAI misconduct behaviors.

*Psychopathy* is characterized by impulsivity, low empathy, and a propensity for unethical behavior^35^. Individuals exhibiting high levels of psychopathy often display a lack of remorse, shallow affect, and a disregard for the impact of their actions on others^35,41^. These traits may contribute to ethical indifference and a greater comfort with rule-breaking, particularly in contexts where personal gain is perceived. In the realm of academic research, such individuals may be more inclined to exploit GAI technologies for competitive advantage, irrespective of ethical considerations. Their impulsive nature and focus on self-interest can lead to a higher likelihood of engaging in misconduct behaviors involving GAI. Therefore, it is hypothesized that:

##### H15

Students higher in psychopathy will report more positive attitudes toward GAI.

##### H16

Students higher in psychopathy will report a higher likelihood of engaging in GAI misconduct behaviors.

*Narcissism* is characterized by grandiosity, a need for admiration, and a lack of empathy^35^. Individuals exhibiting high levels of narcissism often engage in self-enhancing behaviors and seek recognition, sometimes at the expense of ethical considerations. In academic contexts, such traits may drive researchers to utilize GAI technologies to polish their outputs or gain recognition, even through misconduct^42^. This inclination is supported by studies indicating that narcissism is positively correlated with academic dishonesty and unethical behavior^43^. Therefore, it is hypothesized that:

##### H17

Students higher in narcissism will report more positive attitudes toward GAI.

##### H18

Students higher in narcissism will report a higher likelihood of engaging in GAI misconduct behaviors.

## Study 1

The primary aim of Study 1 was to develop a psychometrically robust scale specifically designed to measure students’ attitudes toward the use of GAI in academic contexts. The scale development process was guided by three core principles: (a) the scale should be unidimensional to enable clear interpretation of overall attitude scores; (b) it should incorporate items representing the three classic components of attitudes in psychology—cognitive, affective, and behavioral; and (c) it should contain both positively and negatively worded items to capture the full evaluative spectrum, while mitigating agreement bias.

Grounded in social psychological theories of attitude structure^44^ and informed by existing general AI attitude measures^3,5^, we initially generated 24 items, encompassing cognitive, affective, and behavioral components. Drawing upon established AI attitude measures, such as the General Attitudes Toward Artificial Intelligence Scale^3^ and the ATTARI-12^5^, a pool of 24 items was generated—eight items per component—to comprehensively capture students’ evaluations of generative AI in academic contexts. Within each dimension, four items expressed positive attitudes and four expressed negative attitudes, ensuring conceptual balance and minimizing acquiescence bias.

A positive attitude reflects beliefs that GAI enhances academic efficiency, creativity, and productivity (e.g., “Using GAI helps me think more critically during the writing process”, “I find GAI use in academia inspiring and intellectually stimulating”). A negative attitude reflects ethical or reliability concerns (e.g., “GAI tools are unreliable and produce misleading academic content”, “GAI makes me feel uncomfortable about the future of academic integrity”). All items were rated on a 5-point Likert scale (1 = Strongly disagree, 5 = Strongly agree). Negatively worded items were reverse-scored so that higher total scores indicated a more favorable overall attitude toward the academic use of GAI.

In the second stage, a panel of three researchers—whose expertise covered educational assessment, psychology, and educational technology—reviewed the initial item pool. Items were revised or eliminated if they were semantically redundant, too contextually narrow, or ambiguous in focus. This refinement process resulted in a 12-item scale with each attitudinal component (cognitive, affective, behavioral) represented by four items: two positively and two negatively worded. Although the items span distinct psychological dimensions, they were theorized to load onto a single latent factor reflecting an individual’s general attitude toward the use of GAI in academic work.

To assess construct validity, Study 1 also included measures of participants’ intention to use GAI and actual use of GAI in academic contexts. We expected that general attitudes measured by the GAI Attitudes Scale would correlate positively with their intention and actual use of GAI, based on Theory of Planned Behavior^45^. Additionally, to evaluate potential susceptibility to social desirability bias, we included a short-form social desirability scale^46^. Given the careful phrasing and balanced item valence, we hypothesized that GAI attitudes scores would not be significantly associated with socially desirable responding.

### Methods

#### Ethics statement

This research received ethical approval from Peking University Institutional Review Board. All methods were performed in accordance with relevant guidelines and regulations. Informed consent was obtained from all participants prior to their participation in the study, and they were assured of their anonymity and the voluntary nature of their involvement.

### Instruments

#### GAI Attitudes Scale

We administered the newly developed GAI Attitudes Scale to assess participants’ attitudes toward GAI in academic settings. The scale includes 12 items representing the cognitive, affective, and behavioral components of attitudes, each balanced with positive and negative wording. Responses were recorded using a five-point Likert scale (1 = strongly disagree, 5 = strongly agree). Psychometric properties and descriptive statistics are reported in the Results section.

#### Behavioral intention to use GAI in academic contexts

Participants’ intention to use generative AI in academic settings was measured with a single-item indicator adapted for this study. The item asked: “To what extent would you like to use generative AI (e.g., ChatGPT, Claude, Gemini) in your academic work (e.g., research, writing, teaching)?” Responses were recorded on a 5-point Likert scale ranging from 1 (Not at all) to 5 (Very strongly intend to).

#### Actual use of GAI in academic settings

To assess participants’ current engagement with generative AI tools in academic domains, a single item was used: “How frequently do you currently use generative AI tools (e.g., ChatGPT, Claude, Gemini) in your academic work (e.g., writing papers, preparing lectures, analyzing data)?” Responses were measured on a 5-point scale ranging from 1 (Never) to 5 (Very frequently).

#### Social desirability

To assess potential response bias, we included a 17-item Social Desirability Scale^19^. Participants responded to whether a set of socially desirable or undesirable behaviors described them (true/false format). Scores ranged from 0 to 17, with higher scores indicating greater tendency toward socially desirable responding.

#### Exclusion criteria

To ensure participant attentiveness and response validity, two attention check items were embedded in the questionnaire at different stages. These checks were designed as conceptual comprehension questions rather than direct instruction-following items, ensuring that participants were actively reading and understanding the content rather than mechanically clicking through. The first check (Q5) was a simple factual question (“Which of the following is a fruit?”) designed to detect random or inattentive responding. The second check (Q107) appeared near the end of the survey (“What is the main theme of this questionnaire?”) and served to confirm that participants had understood the overall purpose of the study. Participants who failed either attention check or completed the survey in less than 120 s—a threshold established through pilot testing—were excluded from the final dataset.

#### Participants

To ensure sufficient statistical power for scale validation and subsequent correlational analyses, a priori power analysis conducted using semPower (Version 2.0.1) indicated that a minimum sample size of 500 participants was necessary.

A total of 625 participants, students from five universities in China, were recruited via Wenjuanxing website, which is a popular website for collecting survey responses in China. Incentives of 5 RMB were provided for each completed questionnaire. The average completion time was approximately 4 min. Based on the exclusion criteria, 78 participants were excluded (31 for completion time, 47 for failing the description task), yielding a final sample of 547 participants (279 female, 268 male). Participants ranged in age from 20 to 35 years.

After providing informed consent, participants were first asked to create a unique anonymous identifier by combining two elements only they would know—for example, the name of an elementary school teacher and the month of their birth. This identifier could not be traced back to participants’ identities by the researchers but allowed for accurate matching in the follow-up test–retest reliability assessment.

Participants first answered demographic questions, followed by an attention check question. Next, they completed the GAI Attitudes Scale, which assessed their attitudes toward the use of GAI in academic contexts. They then responded to measures evaluating their intention to use GAI, actual GAI usage, a social desirability scale, and a final attention check in the form of a summary question.

##### Data analysis

All statistical analyses for Study 1 were performed using SPSS 28.0 and Mplus 8.8. Prior to analysis, data were screened for missing values and outliers. Descriptive statistics were computed for all variables, and the internal consistency of each subscale was examined using Cronbach’s α coefficients.

To assess the factorial validity of the 12-item GAI Attitudes Scale, we conducted a series of confirmatory factor analyses (CFAs) testing alternative measurement models, including single-factor, three-factor, and bifactor S-1 models. Model fit was evaluated using the χ^2^/df ratio, Comparative Fit Index (CFI), Tucker–Lewis Index (TLI), Root Mean Square Error of Approximation (RMSEA), and Standardized Root Mean Square Residual (SRMR), following Hu and Bentler (1999).

Correlation analyses were performed to examine relationships among demographic variables and attitudinal dimensions, which also served to identify potential control variables for subsequent analyses. Independent-samples t-tests and one-way ANOVAs were used, where appropriate, to test for mean differences across gender and discipline. All tests were two-tailed, and statistical significance was set at* p* < 0.05.

### Results

#### Psychometric analysis of the GAI Attitudes Scale

Before addressing the main hypotheses, we first evaluated the psychometric properties of the 12-item GAI Attitudes Scale. A series of confirmatory factor analyses (CFAs) were conducted to examine the factorial structure, including a single-factor model, a three-factor model (cognitive, affective, behavioral), and a bifactor S-1 model. Model fit indices (χ^2^/df, CFI, TLI, RMSEA, SRMR) were assessed according to Hu and Bentler’s (1999) criteria. Reliability was evaluated using Cronbach’s α coefficients for each sub-dimension and for the overall scale.

We assessed the factorial validity of the scale based on the assumption that all items continue to reflect a single underlying construct—students’ attitudes toward GAI. To evaluate this, we conducted a confirmatory factor analysis comparing a series of models with progressively fewer constraints. As shown in Table 1, among the tested models, the bifactor S-1 model with content facets (Model b) demonstrated the best overall fit. It significantly outperformed the single-factor model (Model a), as indicated by the chi-square difference test (Δχ^2^(8) = 20.02, *p* = 0.010), and showed improved CFI, RMSEA, SRMR, and lower AIC/BIC values. While the full model including both content and wording factors (Model d) had slightly better absolute fit indices (e.g., lowest RMSEA and SRMR), the improvement over Model (b) was not statistically significant (*p* = 0.236) and came at the cost of model complexity.

**Table 1:** Goodness of fit for competing confirmatory factor models for the GAI Attitudes Scale.

		χ^2^	df	CFI	RMSEA	SRMR	AIC	BIC	Comp	Δχ^2^	Δdf	p
(a)	Single factor model	66.89	54	0.96	0.020	0.021	15,574.25	15,642.12	–	–	–	–
Bifactor S-1 models with one global factor and orthogonal specific factors for …												
(b)	Content facets	46.87	46	0.97	0.006	0.0175	15,568.89	15,664.14	a	20.02	8	.010^*^
(c)	Item wording	60.33	48	0.96	0.021	0.0200	15,571.88	15,659.02	a	6.56	6	.365
(d)	Content facets and item wording	38.84	40	0.95	0.005	0.0157	15,567.73	15,679.33	b	8.03	6	.236
									c	21.49	8	0.129

**Fig. 1:**
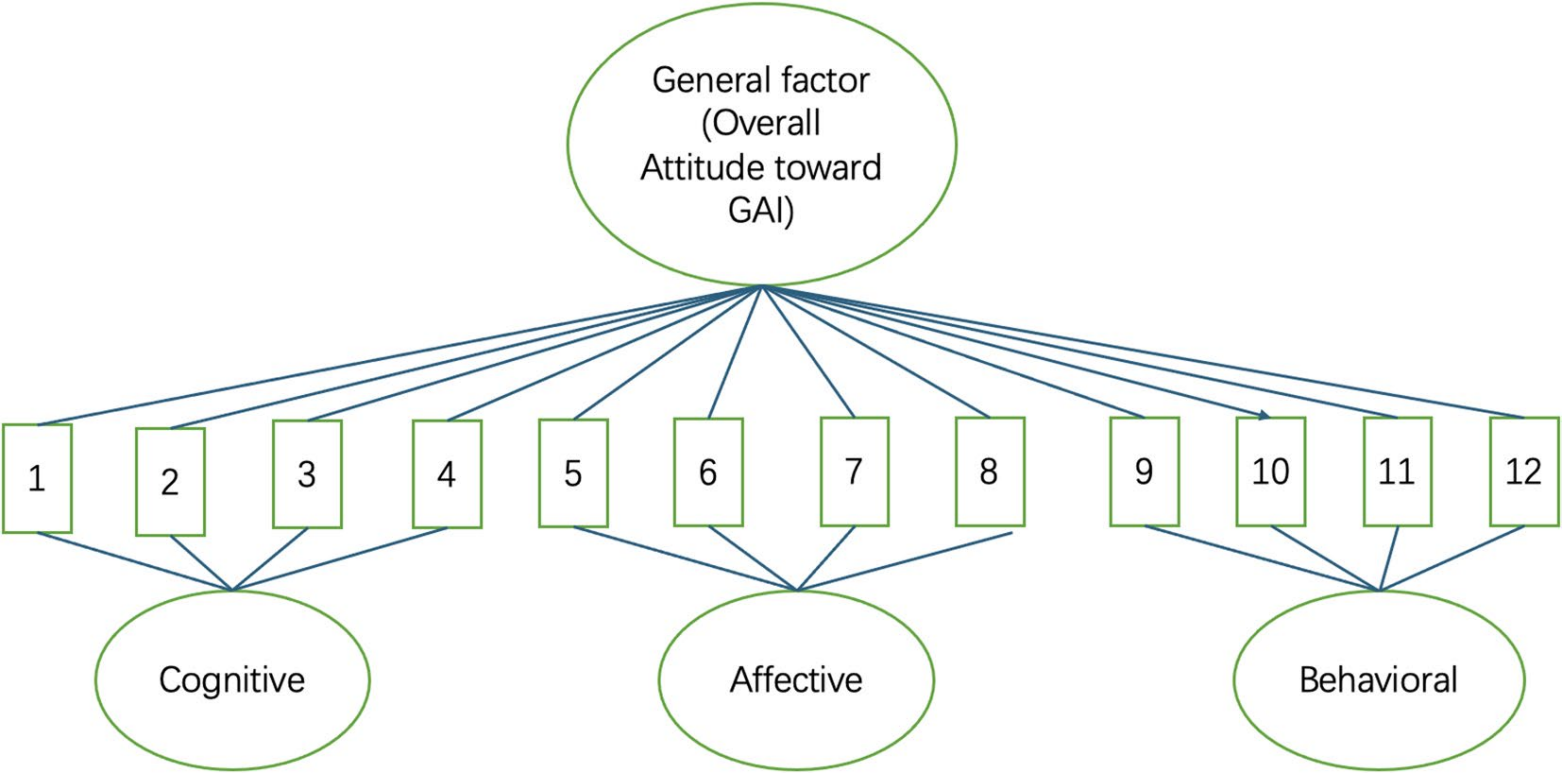
Bifactor S-1 model of the GAI Attitudes Scale. *Note*: 1–12 represents 12 items in the scale.

To illustrate the factorial structure of the GAI Attitudes Scale, Fig. 1 presents the bifactor S-1 model with content facets. The model specifies one general factor representing overall attitudes toward generative AI and three orthogonal specific factors corresponding to the cognitive, affective, and behavioral dimensions. Each of the twelve items loads on both the general factor and its respective specific factor, capturing shared and unique variance across the content domains.

As shown in Table 2, all 12 items of the GAI Attitudes Scale demonstrated moderate to strong standardized loadings on the general factor (range = 0.58 to 0.72), supporting the presence of a common underlying construct—attitudes toward GAI in academic contexts. Items from the cognitive (Items 1–4) and affective (Items 5–8) subdomains also showed meaningful loadings on their respective specific factors (range = 0.27 to 0.38), indicating content-specific variance beyond the general factor. Residual variances ranged from 0.30 to 0.49, and bifactor indices were computed to further evaluate the influence of multidimensionality. The general factor accounted for 86% of the common variance (ECV = 0.86), while the specific factors contributed only marginally. The omega hierarchical coefficient for the general factor was 0.92, indicating that the majority of the reliable variance in total scores can be attributed to the general construct—students’ overall attitudes toward GAI. Taken together, these results support the interpretation of the scale as an essentially unidimensional measure, justifying the use of a total score.

**Table 2:** Factor loading pattern for the GAI attitudes scale.

Item	Standardized factor loadings (on general factor)	Standardized factor loadings (on specific factor)	Residual variance
1	0.62	0.31 (Cognitive)	0.42
2	0.58	0.29 (Cognitive)	0.49
3	0.65	0.33 (Cognitive)	0.38
4	0.59	0.36 (Cognitive)	0.41
5	0.68	0.27 (Affective)	0.35
6	0.61	0.34 (Affective)	0.42
7	0.70	0.29 (Affective)	0.31
8	0.63	0.38 (Affective)	0.37
9	0.72	–	0.30
10	0.66	–	0.37
11	0.69	–	0.34
12	0.60	–	0.44

Items 1–4 represent the cognitive subdimension; items 5–8 the affective subdimension; and items 9–12 the behavioral subdimension.

Descriptive statistics and reliability estimates for the study variables are presented in Table 3. The internal consistency of the GAI Attitudes Scale was excellent (Cronbach’s α = 0.92). The distribution approximated a near-normal distribution (Skew = –0.08, Kurtosis = 0.02). The social desirability scale demonstrated acceptable reliability (α = 0.79). Participants reported high behavioral intention (M = 4.18, SD = 0.51) and actual (M = 4.18, SD = 0.50) use of GAI tools, with the two items showing high internal consistency when combined (α = 0.93).

**Tabl﻿e 3:** Descriptive statistics and correlation analysis.

							1	2	3	4	5	6
		Cronbach’s α	M	SD	Skew	Kurt	t(p)					
1	GAI Attitudes Scale	0.916	50.22	4.9	−0.08	0.02						
2	Social Desirability	0.794	13.63	3.27	−1.17	0.82	−0.03 (0.468)					
3	GAI Intention		4.18	0.51	0.25	0.21	0.86 (0.0)	−0.053 (0.199)				
4	GAI use behavior		4.18	0.5	0.31	0.28	0.853 (0.0)	−0.047 (0.246)	0.863 (0.0)			
5	Age		37.66	10.61	−0.02	−1.25	0.04 (0.325)	0.026 (0.518)	0.018 (0.657)	0.06 (0.142)		
6	Gender						0.117 (0.004)	−0.016 (0.697)	0.115 (0.005)	0.103 (0.011)	−0.006 (0.879)	
7	Degree						0.007 (0.868)	0.008 (0.854)	−0.032 (0.436)	−0.033 (0.427)	−0.036 (0.382)	−0.014 (0.724)

Prior to regression analysis, correlations among demographic variables (Age, Gender, and Degree) and the main study variables were examined to identify significant covariates. Variables that showed significant associations with the outcome variable (GAI attitudes) were entered as control variables in Step 1 of the hierarchical regression model. Pearson correlation analyses revealed strong positive associations between attitudes toward GAI and both behavioral intention (r = 0.86, *p* < 0.001) and actual use (r = 0.85,* p* < 0.001). Intention and use were also highly correlated (r = 0.86,* p* < 0.001), supporting the convergent validity of the measures. Social desirability showed no significant correlation with GAI attitudes (r = –0.03,* p* = 0.468) or use (r = –0.05,* p* = 0.246), suggesting minimal response bias. Gender was weakly but significantly associated with attitudes (r = 0.12,* p* = 0.004), intention (r = 0.12,* p* = 0.005), and use (r = 0.10,* p* = 0.011), with males scoring slightly higher. Age and degree level were not significantly related to any GAI-related variables.

#### Test–retest reliability

In order to further evaluate the reliability of the GAI Attitudes Scale by assessing its test–retest reliability, the scale was administered to the same participants—postgraduate students from five universities in China—a second time. The instructors at these universities were re-contacted to help administer the second round of the survey to the same groups of students. A total of 383 participants completed the GAI Attitudes Scale for a second time, allowing for the assessment of test–retest reliability. The survey was conducted via the Wenjuanxing platform, and responses were matched using the unique anonymous identifiers provided by participants. Of the 383 responses, 381 could be matched to the initial survey. Among these, 3 responses were excluded due to incompleteness, resulting in 378 valid cases for the test–retest reliability analysis.

The internal consistency reliability of the GAI Attitudes Scale was also high this time (Cronbach’s α = 0.912), indicating strong scale coherence over time. Descriptive statistics confirmed that the distribution of scores approximated normality, with acceptable levels of skewness (–0.319) and kurtosis (0.24). GAI Attitudes Scale scores followed a reasonably symmetrical and mesokurtic distribution at both measurement points, consistent with expectations for a psychometrically sound scale. Most importantly, the test–retest reliability was strong, with a Pearson correlation of r(378) = 0.856,* p* < 0.001, demonstrating that participants’ attitudes toward GAI in academic contexts were highly stable over time.

##### Discussion on results of Study 1

The results confirmed that the newly developed GAI Attitudes Scale demonstrates strong internal consistency, test–retest reliability, and factorial validity. This finding supports the theoretical assumption that students’ evaluations of GAI—whether positive or negative—are largely governed by a unified latent dimension reflecting their overall acceptance or skepticism of AI-based academic tools. The strong correlations between attitude scores and both behavioral intention and actual use of GAI further establish convergent validity, consistent with the Theory of Planned Behavior, which posits that positive attitudes foster stronger behavioral intentions^45,47,48^. The absence of a significant correlation between attitudes and social desirability also suggests that responses were not substantially biased by impression management.

Overall, the results indicate that the GAI Attitudes Scale is a valid and reliable measure that can be confidently applied in future studies investigating users’ psychological responses to generative AI. The unidimensional nature of the scale simplifies interpretation while preserving theoretical richness through the inclusion of cognitive, affective, and behavioral content. This validation provides an essential foundation for subsequent research—such as Study 2—that explores the antecedents and consequences of these attitudes.

## Study 2

Building on the initial validation of the GAI Attitudes Scale in Study 1, Study 2 addressed the second core research objective: examining how individual differences in personality traits predict students’ attitudes toward GAI in academic contexts, as well as their GAI-related misconduct behaviors. Specifically, this study focused on two complementary personality frameworks—the HEXACO Personality Inventory and the Dark Triad traits. To promote transparency, we preregistered Study 2 prior to data collection, outlining all hypotheses and the intended analyses (https://aspredicted.org/93ny-7qhg.pdf).

### Methods

#### Participants and data quality control

An a priori power analysis conducted using G^*^Power (assuming a small to moderate effect size of f^2^ = 0.08, with 80% power, α = 0.05, and 12 predictors in a hierarchical linear regression) indicated that a minimum sample size of 234 participants was required.

To ensure adequate power and allow for potential exclusions during data screening, we recruited a total of 1007 participants via the Wenjuanxing platform. Several quality control procedures were implemented during data screening to ensure data integrity and minimize the impact of careless or inattentive responding. First, the questionnaire consisted of 107 items, with most being 5-point Likert scale questions. The expected completion time was between 4 and 8 min, but the data shows that many participants completed the survey between 3 and 4 min. As a result, we set the cutoff time at 3 min, removing 99 responses. Additionally, two attention check questions (Q5 and Q107) were included to assess participant attentiveness. For Q5, which asked, “Which of the following is a fruit?”, responses that incorrectly answered option 1 (*n* = 4) and option 3 (*n* = 2) were excluded, resulting in 6 removals. For Q107, which asked, “What is the main theme of this questionnaire?”, incorrect responses (option 1, *n* = 30 and option 3, *n* = 8) led to the removal of 38 responses. In total, 143 responses were excluded, leaving a final sample of 864 valid responses for analysis.

The final sample had a mean age of 23.1 years (SD = 2.92), and was composed of 558 females, 306 males. The distribution of participants’ level of study is as follows: 50.3% (*n* = 434) were undergraduates, 38.5% (*n* = 333) were master’s students, and 11.2% (*n* = 97) were doctoral students. Among the five disciplinary categories, the highest number of participants were from the “Humanities and Social Sciences” category, with 417 participants, accounting for 48.3% of the sample. The second largest group was from the “Medical and Health Sciences” category, with 140 participants, making up 16.2% of the sample, while the “Business and Economics” category had the fewest participants, with 77, equating to 8.9%.

### Research instruments

#### HEXACO personality inventory scale (Hexaco-60)

Participants’ broad personality traits were measured using the 60-item HEXACO Personality Inventory – Revised (HEXACO-60)^22^. This instrument assesses six core dimensions: Honesty–Humility (e.g., “I wouldn’t use flattery to get a raise or promotion at work”), Emotionality (e.g., “I sometimes can’t help worrying about little things”), Extraversion (e.g., “I feel reasonably satisfied with myself overall”), Agreeableness (e.g., “People sometimes tell me that I am too critical of others”), Conscientiousness (e.g., “I plan ahead and organize things to avoid scrambling at the last minute”), and Openness to Experience (e.g., “I enjoy looking at maps of different places”). Participants responded on a 5-point Likert scale ranging from 1 (strongly disagree) to 5 (strongly agree). Internal consistency was acceptable to excellent across the six dimensions (Honesty–Humility: α = 0.92; Emotionality: α = 0.91; Extraversion: α = 0.84; Agreeableness: α = 0.90; Conscientiousness: α = 0.91; Openness: α = 0.83).

#### Short Dark Triad scale

We assessed participants’ Dark Triad personality traits with the Short Dark Triad scale^19^. This instrument includes nine items on Machiavellianism (e.g., “It’s not wise to tell your secrets”), six items on psychopathy (e.g., “People often say I’m out of control”), and nine items on narcissism (e.g., “Many group activities tend to be dull without me”). Each item was rated on a 5-point Likert-type scale (1 = strongly disagree, 5 = strongly agree). Reliability analyses suggested good to very good internal consistencies for all three scales (Machiavellianism: Cronbach’s α = 0.86; psychopathy: Cronbach’s α = 0.83; narcissism: Cronbach’s α = 0.83).

#### GAI Attitudes Scale

Participants’ attitudes toward GAI were assessed using the Chinese version of the newly developed GAI Attitudes Scale in Study 1. Each item was rated on a 5-point Likert-type scale (1 = strongly disagree, 5 = strongly agree). In the present sample (*N* = 864), the scale demonstrated excellent internal consistency (Cronbach’s α = 0.85).

#### GAI Misconduct Behavior Scale

In this study, we employed a GAI academic misconduct scale adapted from a previously validated instrument developed by Sun et al.^17^, designed to measure how frequently students engage in academic misconduct involving generative AI. To enhance its comprehensiveness, we added a new item to the original four-question scale: “I have used AI to answer in unauthorized exams or tests.” Therefore, the scale consists of five items, each evaluated on a 5-point Likert scale ranging from 1 (never) to 5 (always). In the present study, this instrument exhibited high internal reliability (Cronbach’s α = 0.78). Confirmatory factor analysis supported its unidimensional structure, indicating good model fit (χ^2^(2) = 4.083,* p* = 0.130; CFI = 0.976; RMSEA = 0.036; SRMR = 0.004).

##### Data analysis

Statistical analyses for Study 2 were conducted using SPSS 28.0. Prior to the main analyses, data were screened for missing values, normality, and influential cases. Assumption checks confirmed that the residuals were independent and normally distributed, with no evidence of multicollinearity or heteroskedasticity. Cook’s distance values further indicated the absence of influential outliers.

Descriptive statistics and zero-order correlations among all variables were first computed to examine preliminary relationships and to identify potential control variables (age, gender, level of study, and discipline) for regression analyses.

Hierarchical linear regression analyses were then performed to examine the predictive roles of the HEXACO personality dimensions, the Dark Triad traits, and attitudes toward GAI in explaining two outcome variables: (a) attitudes toward GAI and (b) GAI-related academic misconduct behaviors. Predictors were entered in sequential steps to determine their incremental contribution to explained variance (R^2^). Specifically, demographic variables were entered in Step 1, HEXACO dimensions in Step 2, Dark Triad traits in Step 3, and GAI attitudes (for the misconduct model only) in Step 4.

The change in explained variance (ΔR^2^) at each step and standardized regression coefficients (β) were examined to assess the relative predictive power of each construct. All statistical tests were two-tailed, and significance was set at* p* < 0.05.

### Results

#### Descriptive statistics and correlations

Table 4 presents the descriptive statistics (means, standard deviations, skewness, and kurtosis) and bivariate Pearson correlations among the study variables. The sample (*N* = 864) had a mean age of 23.14 years (SD = 2.92). Average scores on the HEXACO dimensions ranged from 2.71 (Neuroticism) to 3.45 (Extraversion), while the Dark Triad traits ranged from 2.44 (Psychopathy) to 2.99 (Narcissism). The mean score for attitudes toward GAI was 3.33 (SD = 0.79), and for GAI academic misconduct was 1.79 (SD = 0.80).

**Table 4:** Descriptive statistics and correlations.

Variable	1	2	3	4	5	6	7	8	9	10	11	12	13	14	M	SD
1. Age															23.1	2.92
2. Gender	0.02															
3. Level of study	−0.03	0.11^**^														
4. Disciplines	−0.11^***^	−0.04	−0.42^***^													
5. Honesty–Humility	0.04	0.01	0.04	−0.03											3.41	0.55
6. Emotionality	−0.02	0.00	−0.03	0.01	−0.01										3.25	0.76
7. Extraversion	0.05	−0.03	−0.01	0.01	−0.02	0.01									3.2	0.72
8. Agreeableness	−0.02	0.05	−0.01	−0.02	0.18^***^	−0.01	0.09^*^								3.45	0.89
9. Openness to Experience	0.04	−0.01	−0.02	0.00	−0.04	−0.02	0.15^***^	−0.04							3.39	0.56
10. Neuroticism	0.00	0.05	0.02	−0.04	0.15^***^	0.00	0.04	0.13^***^	0.11^**^						3.42	0.72
11. Machiavellianism	0.00	−0.01	0.00	−0.03	−0.27^***^	−0.01	−0.03	−0.24^***^	0.04	0.04					2.71	0.66
12. Psychopathy	0.02	−0.02	−0.07^*^	0.04	−0.25^***^	0.01	0.10^**^	−0.20^***^	0.05	−0.17^***^	0.27^***^				2.44	0.52
13. Narcissism	0.02	0.04	−0.04	0.05	−0.12^***^	0.04	0.03	−0.01	0.15^***^	0.01	0.18^***^	0.14^***^			2.99	0.82
14. GAI attitudes	0.02	0.01	0.02	0.00	−0.08^*^	0.01	0.17^***^	−0.02	0.18^***^	−0.06	−0.06	0.01	0.09^**^		3.3	0.79
15. GAI misconduct	0.05	−0.02	−0.01	0.00	−0.38^***^	0.00	−0.02	−0.30^***^	0.01	−0.25^***^	0.33^***^	0.37^***^	0.12^***^	0.11^**^	1.79	0.8

*N* = 864.* p* < 0.05^*^,* p* < 0.01^**^,* p* < 0.001^***^Gender is coded as: 1 = Male; 2 = Female. Level of study is coded as: 1 = Undergraduate; 2 = Master’s; 3 = Doctoral. Disciplines are coded as: 1 = Humanities and Social Sciences; 2 = Science and Engineering; 3 = Business and Economics; 4 = Medical and Health Sciences; 5 = Other.

Attitudes toward GAI demonstrated positive correlations with Extraversion (r = 0.17,* p* < 0.001), Agreeableness (r = 0.18,* p* < 0.001), and Neuroticism (r = 0.11,* p* < 0.01), implying that more outgoing, cooperative, and emotionally sensitive students tended to express more favorable views of GAI. A small negative correlation with Openness to Experience (r = –0.08,* p* < 0.05) suggested that students who are highly open and intellectually curious might approach GAI use with greater caution and critical evaluation.

GAI academic misconduct showed positive correlations with Machiavellianism (r = 0.33,* p* < 0.001), Psychopathy (r = 0.37,* p* < 0.001), Narcissism (r = 0.12,* p* < 0.001), and Neuroticism (r = 0.25,* p* < 0.001), indicating that individuals high in manipulative, impulsive, or self-centered tendencies were more prone to unethical use of GAI. Conversely, GAI misconduct was negatively correlated with attitudes toward GAI (r = –0.25,* p* < 0.001), Emotionality (r = –0.02,* p* < 0.05), and Openness to Experience (r = –0.06,* p* < 0.05), suggesting that students who held more positive attitudes toward GAI, were more emotionally responsive, or demonstrated higher intellectual openness were less likely to engage in AI-related academic misconduct.

#### The predictive role of HEXACO personality inventory and the Dark Triad on GAI attitudes

A hierarchical linear regression analysis was conducted to examine the predictors of participants’ attitudes toward GAI (see Table 5). The analysis proceeded in three steps.

**Table 5:** Hierarchical linear regression predicting GAI attitudes.

Predictor	β	*t*	*p*	R^2^	ΔR^2^
**Step 1**				0.001	–
Gender	0.004	0.90	0.369		
Age	0.013	0.45	0.656		
Level of study	0.005	0.58	0.564		
Discipline	0.002	0.24	0.810		
**Step 2**				0.126	0.125
Honesty–Humility	0.011	1.70	0.089		
Emotionality	0.036	0.52	0.602		
Extraversion	0.248	4.51	< 0.001^***^		
Agreeableness	0.014	0.43	0.669		
Conscientiousness	0.038	0.72	0.471		
Openness to Experience	0.358	6.12	< 0.001^***^		
**Step 3**				0.359	0.233
Machiavellianism	0.253	4.86	< 0.001^***^		
Narcissism	–0.002	–0.23	0.814		
Psychopathy	–0.004	–0.62	0.535		

*N* = 864. Dependent variable: GAI attitudes.* p* < 0.05;* p* < 0.01;* p* < 0.001. Control variables entered at Step 1: gender, age, level of study, discipline.

Step 1: Gender, age, level of study, and disciplines were entered as predictors. The model accounted for a negligible amount of variance in GAI attitudes (R^2^ = 0.001). None of them (e.g., age (β = 0.013,* p* = 0.656), gender (β = 0.004,* p* = 0.369)) emerged as significant predictors.

Step 2: In the second step, the six HEXACO personality dimensions were added, resulting in a notable increase in explained variance (ΔR^2^ = 0.125). Among these, Honesty–Humility showed a marginal effect (β = 0.011,* p* = 0.089), while Extraversion (β = 0.248,* p* < 0.001) and Openness to Experience (β = 0.358,* p* < 0.001) emerged as significant positive predictors of GAI attitudes.

Step 3: The final step introduced the Dark Triad personality traits, which further increased the explained variance (ΔR^2^ = 0.233). Of these, Machiavellianism was a significant positive predictor (β = 0.253,* p* < 0.001), whereas Narcissism (β = -0.002,* p* = 0.814) and Psychopathy (β = -0.004,* p* = 0.535) were not significant.

Overall, HEXACO Extraversion and Openness to Experience, as well as Machiavellianism from the Dark Triad, emerged as robust positive predictors of GAI attitudes. In contrast, age, gender, level of study, disciplines and other personality traits were not significant predictors.

#### The predictive role of HEXACO personality inventory and the Dark Triad on GAI misconduct behaviors

A hierarchical linear regression analysis was conducted to examine the predictive roles of the HEXACO personality dimensions, Dark Triad traits, and attitudes toward GAI in GAI-related academic misconduct behaviors (see Table 6).

**Table 6:** Hierarchical linear regression predicting GAI misconduct behaviors.

Predictor	β	*t*	*p*	R^2^	ΔR^2^
**Step 1**				0.002	–
Gender	0.006	0.81	0.421		
Age	–0.011	–0.32	0.749		
Level of study	–0.003	–0.45	0.658		
Discipline	–0.005	–0.51	0.610		
**Step 2**				0.236	0.234
Honesty–Humility	–0.36	–6.78	< 0.001^***^		
Emotionality	–0.04	–0.82	0.414		
Extraversion	–0.05	–1.04	0.299		
Agreeableness	–0.15	–3.92	< 0.001^***^		
Conscientiousness	–0.25	–4.86	< 0.001^***^		
Openness to Experience	0.02	0.44	0.660		
**Step 3**				0.494	0.258
Machiavellianism	0.07	1.12	0.262		
Narcissism	0.24	4.81	< 0.001^***^		
Psychopathy	0.25	4.73	< 0.001^***^		
**Step 4**				0.498	0.004
GAI attitudes	–0.02	–0.60	0.550		

*N* = 864. Dependent variable: GAI misconduct behaviors.* p* < 0.05;* p* < 0.01;* p* < 0.001.

Step 1: In the first step, gender, age, level of study, and disciplines were included as predictors. These variables explained only a negligible portion of variance in GAI misconduct (R^2^ = 0.002), with none demonstrating a significant effect.

Step 2: The addition of the six HEXACO personality dimensions in the second step led to a substantial improvement in model fit (ΔR^2^ = 0.234,* p* < 0.001). Honesty–Humility (β =  − 0.36,* p* < 0.001), Agreeableness (β =  − 0.15,* p* < 0.001), and Conscientiousness (β =  − 0.25,* p* < 0.001) all emerged as significant negative predictors of GAI misconduct.

Step 3: Incorporating the Dark Triad traits in the third step further improved the explanatory power of the model (ΔR^2^ = 0.258,* p* < 0.001). Both Narcissism (β = 0.24,* p* < 0.001) and Psychopathy (β = 0.25,* p* < 0.001) were significant positive predictors of GAI misconduct, whereas Machiavellianism was not significant.

Step 4: In the final step (Step 4), GAI attitudes were entered into the regression model after controlling for HEXACO and Dark Triad personality traits. The inclusion of this variable did not significantly increase the explained variance in GAI misconduct behaviors (ΔR^2^ = 0.004,* p* = 0.55; final R^2^ = 0.498). The standardized regression coefficient for GAI attitudes was non-significant (β = –0.02,* p* = 0.55), indicating that attitudes toward GAI did not provide incremental explanatory power beyond the effects of personality traits.

The final regression model accounted for a substantial proportion of the variance in GAI misconduct behaviors (R^2^ = 0.498). These findings indicate that personality traits—particularly lower levels of Honesty–Humility, Agreeableness, and Conscientiousness, and higher levels of Narcissism and Psychopathy—are robust predictors of GAI-related academic misconduct. In contrast, after accounting for these personality dimensions, attitudes toward GAI provided no additional explanatory power.

#### Summary of results

##### Predictors of GAI attitudes

The hypotheses related to the HEXACO personality traits were partially supported. Extraversion and Openness to Experience emerged as significant positive predictors of GAI attitudes. Therefore, H5 and H11 were supported. However, the remaining personality traits did not show statistically significant effects, and thus H1, H3, H7, and H9 were not supported. Regarding the Dark Triad, only Machiavellianism emerged as a significant positive predictor of GAI attitudes, supporting H13. In contrast, Narcissism and Psychopathy did not show significant effects. Therefore, H15 and H17 were not supported.

#### Predictors of GAI misconduct behaviors

In terms of GAI misconduct, the HEXACO traits were more consistently aligned with the hypotheses. Honesty–Humility, Agreeableness, and Conscientiousness all emerged as significant negative predictors of GAI misconduct, supporting H2, H8, and H10. However, the remaining personality traits did not show statistically significant effects, and thus H4, H6 and H12 were not supported. In terms of the Dark Triad traits, both Narcissism and Psychopathy were significant positive predictors of GAI misconduct, supporting H16 and H18. Machiavellianism, however, did not significantly predict GAI misconduct in this analysis, and thus H14 was not supported. In addition, GAI attitudes did not contribute significant additional explanatory power in predicting GAI misconduct behaviors after accounting for personality traits.

##### Discussion on findings of Study 2

The observed associations between personality traits and GAI-related attitudes and behaviors can be interpreted through established personality mechanisms. Individuals high in Extraversion may show more favorable attitudes toward generative AI because their sociability and assertiveness make them more open to experimentation and engagement with novel tools that enhance communication and performance^5,49^. Similarly, those high in Openness to Experience tend to value innovation and intellectual curiosity, leading them to perceive GAI as a stimulating and creative extension of their cognitive repertoire^50,51^. In contrast, students high in Honesty–Humility and Conscientiousness are typically guided by strong internalized moral norms and self-regulatory tendencies^25^, which reduce the likelihood of unethical GAI use. Their behavior aligns with theories of moral identity and self-control, suggesting that individuals with stronger ethical orientations are less susceptible to misconduct temptations when new technologies lower traditional barriers to dishonesty^52^.

Conversely, the positive associations of Narcissism and Psychopathy with GAI-related misconduct may reflect the self-serving and impulsive nature of these traits. Narcissistic individuals, driven by a desire for recognition and superiority, may exploit GAI to enhance their academic outputs and maintain a grandiose self-image, even at the expense of ethical boundaries^19^. Psychopathic tendencies, marked by low empathy and poor behavioral inhibition, can further erode moral restraint, leading to opportunistic or careless misuse of GAI tools. Together, these patterns support a dispositional model in which self-regulation, moral concern, and social orientation operate as key psychological mechanisms underlying ethical or unethical engagement with GAI. These findings extend previous research on technology ethics^12,53,54^ by illustrating how enduring personality structures interact with emerging technological affordances to shape both innovation and misconduct in academic contexts.

Interestingly, the hierarchical regression results revealed that, after controlling for personality traits, attitudes toward GAI no longer contributed significant additional explanatory power in predicting GAI-related academic misconduct (ΔR^2^ = 0.004,* p* = 0.55). This finding suggests that personality traits may exert a more direct and robust influence on unethical academic behaviors than attitudinal dispositions.

Traditionally, the attitude–behavior link has been considered a core assumption in social psychology, as outlined in the Theory of Planned Behavior^45^ and related frameworks, which posit that attitudes predict intentions and subsequent behaviors. However, our results imply that in contexts involving generative AI—a novel, ethically ambiguous technology—deep-seated personality tendencies (e.g., low Honesty–Humility, high Psychopathy) may override attitudinal intentions when individuals decide whether to misuse such tools. This finding aligns with emerging research suggesting that moral decision-making in AI-assisted academic settings is more trait-driven than belief-driven^55,56^.

Theoretically, this challenges the sufficiency of attitudinal models alone for explaining AI-related academic misconduct and underscores the need for integrative models that combine personality frameworks (e.g., HEXACO, Dark Triad) with situational and moral-cognitive variables. Practically, it suggests that interventions focused solely on shaping students’ attitudes toward AI ethics may be insufficient without addressing the underlying personality dispositions that predispose certain individuals to misuse AI tools.

Although the present findings were based on a Chinese sample, they align with research from Western countries showing that Extraversion and Openness to Experience predict more favorable technology attitudes, whereas Honesty–Humility and Conscientiousness are negatively related to unethical behaviors^5,13^. This convergence suggests that the personality–behavior mechanisms shaping responses to generative AI may be broadly cross-cultural. However, cultural factors such as collectivist orientations, social desirability, and differing perceptions of academic integrity in East Asian contexts may influence how individuals express these tendencies in practice. Future studies should employ cross-cultural comparative designs to examine whether these trait–behavior relationships are moderated by cultural values or institutional norms.

## General discussion

Across both studies, this research provides an integrated understanding of how personality traits influence students’ attitudes toward and ethical engagement with GAI. Study 1 developed and validated the GAI Attitudes Scale, confirming its strong reliability and convergent validity, while Study 2 demonstrated that stable personality dispositions, rather than attitudes alone, are stronger predictors of GAI-related misconduct.

Specifically, Extraversion and Openness to Experience were associated with more positive GAI attitudes, reflecting curiosity and receptivity to technological innovation^5,48^. Conversely, Honesty–Humility, Agreeableness, and Conscientiousness negatively predicted GAI misconduct, consistent with prior evidence that moral and prosocial traits discourage unethical behavior^11,12^. In contrast, Narcissism and Psychopathy positively predicted GAI misuse, echoing previous findings linking dark personality traits to dishonest or exploitative technology use^13,25^.

These results extend the Theory of Planned Behavior^45^ and personality-based models of ethical decision-making by showing that while attitudes toward GAI foster willingness to use such tools, enduring personality traits largely determine whether that use remains ethical. Educational interventions should therefore combine attitude-shaping strategies with personality-informed approaches—such as moral reflection and integrity training—to promote responsible GAI engagement in higher education.

## Conclusion

This study examined the predictive roles of HEXACO personality traits and the Dark Triad in shaping students’ attitudes toward GAI and their likelihood of engaging in GAI-related academic misconduct. The findings underscore the significant influence of personality traits on both GAI adoption and ethical behavior within academic contexts.

Specifically, Extraversion and Openness to Experience emerged as positive predictors of favorable GAI attitudes, aligning with existing literature that associates these traits with openness to new technologies and ideas. Conversely, Honesty–Humility did not significantly predict GAI attitudes, suggesting that ethical considerations may not directly influence the adoption of GAI tools in academic settings. Regarding the Dark Triad, Machiavellianism was positively associated with favorable GAI attitudes, indicating that individuals high in this trait may perceive GAI as a strategic tool for personal gain.

In terms of academic misconduct, Honesty–Humility, Agreeableness, and Conscientiousness were significant negative predictors, highlighting the role of ethical and prosocial traits in discouraging unethical behaviors related to GAI. On the other hand, Narcissism and Psychopathy were positive predictors of GAI misconduct, suggesting that individuals high in these traits may exploit GAI for self-serving purposes without regard for ethical standards. Interestingly, Machiavellianism did not significantly predict GAI misconduct, indicating that its influence on unethical behavior may be context-dependent.

These findings contribute to the growing body of literature on the intersection of personality and technology adoption, emphasizing the need for a nuanced understanding of how individual differences influence ethical decision-making in the context of emerging technologies like GAI. Future research should further explore these relationships and consider additional factors such as institutional policies and cultural norms that may mediate the impact of personality on GAI-related behaviors.

## Limitations and future research

First, the study employed a cross-sectional design, which limits the ability to draw causal inferences. The relationships observed between personality traits and GAI-related outcomes could be influenced by other unmeasured variables, such as situational factors or prior experiences with technology. Future research could benefit from using longitudinal designs to track changes in attitudes and behaviors over time and assess the directionality of these relationships.

Second, the study relied on self-report measures, which may be subject to social desirability bias or other response biases. While self-report inventories like HEXACO and the Dark Triad are widely used in personality research, combining them with more objective measures, such as behavioral observations or peer ratings, could provide a more accurate assessment of personality and its influence on GAI-related behaviors. Future studies could consider experimental designs to test the causal effects of personality traits on GAI adoption and misconduct in controlled settings. Additionally, it is worth noting that although the Short Dark Triad scale is widely used for assessing Machiavellianism, narcissism, and psychopathy, previous studies^57–59^ have raised concerns regarding its construct validity and discriminant reliability across different cultural contexts. This limitation should be acknowledged when interpreting the results related to Dark Triad traits in this study.

Third, the sample in this study was limited to university students in China which may not be fully representative of the broader population of technology users. As GAI tools are adopted across various industries, future research should explore how personality traits influence GAI attitudes and behaviors in non-academic contexts. It would be particularly interesting to examine how personality traits interact with professional roles, such as industry professionals, students, or policymakers, to shape their use of GAI.

Finally, while this study focused on personality traits as predictors of GAI attitudes and misconduct, other factors, such as institutional policies, cultural norms, and ethical training, may also play a significant role in shaping GAI-related behaviors. Future research could examine these contextual factors and explore their interaction with personality traits to provide a more holistic understanding of the factors influencing ethical decision-making in the context of emerging technologies.

## Supplementary Information

Below is the link to the electronic supplementary material.

Supplementary Material 1

## Data Availability

The data analyzed in the study are available from the first author upon reasonable request.
