# Supplementary material for: Personality traits measured by the HEXACO personality inventory and the dark triad predict university students’ attitudes and misconduct behaviors related to generative artificial intelligence: Haiying Liang1, Xu Mao2, Michael J. Reiss3*
Source: Sci Rep. 2025 Nov 25;15:41787. doi: 10.1038/s41598-025-25744-4 (PMC12647770; doi:10.1038/s41598-025-25744-4)
Supplement: Supplementary file 1 — Supplementary Material 1 [file 41598_2025_25744_MOESM1_ESM.docx]

**Appendix A**

**Questionnaire used for Study 1**

**English version**

**Gender**

Male

Female

**Age**

**Degree**

Bachelor and below

Master’s

Doctoral

**Attention check**: which of the following is a fruit:

Biscuit

Apple

Cup

**Sub-Scale 1: GAI attitudes scale**

| Item No. | Statement | Dimension |
| --- | --- | --- |
|  | I believe GAI can enhance the quality of academic work. | Cognitive |
|  | GAI tools are unreliable and produce misleading academic content. (R) | Cognitive |
|  | Using GAI helps me think more critically during the writing process. | Cognitive |
|  | I don’t trust GAI tools to support academic research tasks. (R) | Cognitive |
|  | I feel excited about using GAI in my research or teaching. | Affective |
|  | I feel anxious when thinking about integrating GAI into academic work. (R) | Affective |
|  | I find GAI use in academia inspiring and intellectually stimulating. | Affective |
|  | GAI makes me feel uncomfortable about the future of academic integrity. (R) | Affective |
|  | I am likely to incorporate GAI tools in my future academic projects. | Behavioral |
|  | I avoid using GAI because I worry it will negatively affect my work. (R) | Behavioral |
|  | I actively seek out new ways to integrate GAI in my academic workflow. | Behavioral |
|  | I deliberately limit my use of GAI in academic contexts. (R) | Behavioral |

**Behavioral Intention to Use GAI in Academic Contexts**

To what extent would you like to use generative AI (e.g., ChatGPT, Claude, Gemini) in your academic work (e.g., research, writing, teaching)?

Response scale (5-point Likert):

1 – Not at all

2 – Slightly intend to

3 – Moderately intend to

4 – Strongly intend to

5 – Very strongly intend to

**Actual Use of GAI in Academic Contexts**

How frequently do you currently use generative AI tools (e.g., ChatGPT, Claude, Gemini) in your academic work (e.g., writing papers, preparing lectures, analyzing data)?

Response scale (5-point frequency):

1 – Never

2 – Rarely

3 – Occasionally

4 – Often

5 – Very frequently

**Sub-scale 2: The Social Desirability Scale-17 (SDS-17)**

Below you will find a list of statements. Please read each statement carefully and decide if that statement describes you or not. If it describes you, check the word “true”; if not, check the word “false.”

1. I sometimes litter. (R)
2. I always admit my mistakes openly and face the potential negative consequences.
3. In traffic I am always polite and considerate of others.
4. I have tried illegal drugs (for example, marijuana, cocaine, etc.). (R)
5. I always accept others’ opinions, even when they don’t agree with my own.
6. I take out my bad moods on others now and then. (R)
7. There has been an occasion when I took advantage of someone else. (R)
8. In conversations I always listen attentively and let others finish their sentences.
9. I never hesitate to help someone in case of emergency.
10. When I have made a promise, I keep it – no ifs, ands or buts.
11. I occasionally speak badly of others behind their back. (R)
12. I would never live off other people.
13. I always stay friendly and courteous with other people, even when I am stressed out.
14. During arguments I always stay objective and matter-of-fact.
15. There has been at least one occasion when I failed to return an item that I borrowed. (R)
16. I always eat a healthy diet.
17. Sometimes I only help because I expect something in return. (R)

Note: Answer categories are “true” (1) and “false” (0).

**Attention check**: What’s the topic of this study?

Biology

Artificial intelligence

Law

**Appendix B**

**Questionnaire for study 2**

**English version**

**Gender**:

Male

Female

**Age**: ____

**Level of study:**

Undergraduate

Master’s

Doctoral

**Disciplines**

Humanities and Social Sciences

Science and Engineering

Business and Economics

Medical and Health Sciences

Others

Attention check: which of the following is a fruit:

Biscuit

Apple

Cup

**Scale 1 - HEXACO personality (60 items)**

On the following pages, you will find a series of statements about you.

Please read each statement and decide how much you agree or disagree with

that statement. Then indicate your response using the following scale:

5 = strongly agree

4 = agree

3 = neutral (neither agree nor disagree)

2 = disagree

1 = strongly disagree

Please answer every statement, even if you are not completely sure of your

response.

1. I would be quite bored by a visit to an art gallery.
2. I plan ahead and organize things, to avoid scrambling at the last minute.
3. I rarely hold a grudge, even against people who have badly wronged me.
4. I feel reasonably satisfied with myself overall.
5. I would feel afraid if I had to travel in bad weather conditions.
6. I wouldn’t use flattery to get a raise or promotion at work, even if I thought it would succeed.
7. I’m interested in learning about the history and politics of other countries.
8. I often push myself very hard when trying to achieve a goal.
9. People sometimes tell me that I am too critical of others.
10. I rarely express my opinions in group meetings.
11. I sometimes can’t help worrying about little things.
12. If I knew that I could never get caught, I would be willing to steal a million dollars.
13. I would enjoy creating a work of art, such as a novel, a song, or a painting.
14. When working on something, I don’t pay much attention to small details.
15. People sometimes tell me that I’m too stubborn.
16. I prefer jobs that involve active social interaction to those that involve working alone.
17. When I suffer from a painful experience, I need someone to make me feel comfortable.
18. Having a lot of money is not especially important to me.
19. I think that paying attention to radical ideas is a waste of time.
20. I make decisions based on the feeling of the moment rather than on careful thought.
21. People think of me as someone who has a quick temper.
22. On most days, I feel cheerful and optimistic.
23. I feel like crying when I see other people crying.
24. I think that I am entitled to more respect than the average person is.
25. If I had the opportunity, I would like to attend a classical music concert.
26. When working, I sometimes have difficulties due to being disorganized.
27. My attitude toward people who have treated me badly is “forgive and forget.”
28. I feel that I am an unpopular person.
29. When it comes to physical danger, I am very fearful.
30. If I want something from someone, I will laugh at that person’s worst jokes.
31. I’ve never really enjoyed looking through an encyclopedia.
32. I do only the minimum amount of work needed to get by.
33. I tend to be lenient in judging other people.
34. In social situations, I’m usually the one who makes the first move.
35. I worry a lot less than most people do.
36. I would never accept a bribe, even if it were very large.
37. People have often told me that I have a good imagination.
38. I always try to be accurate in my work, even at the expense of time.
39. I am usually quite flexible in my opinions when people disagree with me.
40. The first thing that I always do in a new place is to make friends.
41. I can handle difficult situations without needing emotional support from anyone else.
42. I would get a lot of pleasure from owning expensive luxury goods.
43. I like people who have unconventional views.
44. I make a lot of mistakes because I don’t think before I act.
45. Most people tend to get angry more quickly than I do.
46. Most people are more upbeat and dynamic than I generally am.
47. I feel strong emotions when someone close to me is going away for a long time.
48. I want people to know that I am an important person of high status.
49. I don’t think of myself as the artistic or creative type.
50. People often call me a perfectionist.
51. Even when people make a lot of mistakes, I rarely say anything negative.
52. I sometimes feel that I am a worthless person.
53. Even in an emergency I wouldn’t feel like panicking.
54. I wouldn’t pretend to like someone just to get that person to do favors for me.
55. I find it boring to discuss philosophy.
56. I prefer to do whatever comes to mind, rather than stick to a plan.
57. When people tell me that I’m wrong, my first reaction is to argue with them.
58. When I’m in a group of people, I’m often the one who speaks on behalf of the group.
59. I remain unemotional even in situations where most people get very sentimental.
60. I’d be tempted to use counterfeit money, if I were sure I could get away with it.

Scoring of HEXACO–60 Scales (see Table 1 for Facet-Level Scales):

Honesty-Humility: 6, 12R, 18, 24R, 30R, 36, 42R, 48R, 54, 60R

Emotionality: 5, 11, 17, 23, 29, 35R, 41R, 47, 53R, 59R

Extraversion: 4, 10R, 16, 22, 28R, 34, 40, 46R, 52R, 58

Agreeableness (versus Anger): 3, 9R, 15R, 21R, 27, 33, 39, 45, 51, 57R

Conscientiousness: 2, 8, 14R, 20R, 26R, 32R, 38, 44R, 50, 56R

Openness to Experience: 1R, 7, 13, 19R, 25, 31R, 37, 43, 49R, 55R

(R indicates reverse-scored item.)

**Scale 2 - The Short Dark Triad (24 items)**

Instructions: Please indicate how much you agree with each of the following statements1-5 (Disagree strongly-agree strongly)

*Machiavellianism*

1. It’s not wise to tell your secrets.
2. I like to use clever manipulation to get my way.
3. Whatever it takes, you must get the important people on your side.
4. Avoid direct conflict with others because they may be useful in the future.
5. It’s wise to keep track of information that you can use against people later.
6. You should wait for the right time to get back at people.
7. There are things you should hide from other people to preserve your reputation.
8. Make sure your plans benefit yourself, not others
9. Most people can be manipulated.

*Narcissism*

1. People see me as a natural leader.
2. I hate being the center of attention. (R)
3. Many group activities tend to be dull without me.
4. I know that I am special because everyone keeps telling me so.
5. I like to get acquainted with important people.
6. I feel embarrassed if someone compliments me. (R)
7. I have been compared to famous people.
8. I am an average person. (R)
9. I insist on getting the respect I deserve.

*Psychopathy*

1. I like to get revenge on authorities.
2. I avoid dangerous situations. (R)
3. Payback needs to be quick and nasty.
4. People often say I’m out of control.
5. It’s true that I can be mean to others.
6. People who mess with me always regret it.

**Scale 3 - Attitudes Toward GAI Scale (12 items)**

Instructions: Please indicate the extent to which you agree or disagree with each of the following statements about generative AI (GAI) in academic settings.

Response format:

1 = Strongly Disagree

2 = Disagree

3 = Neutral

4 = Agree

5 = Strongly Agree

*Cognitive Dimension*

1. I believe GAI can enhance the quality of academic work.
2. GAI tools are unreliable and produce misleading academic content. (R)
3. Using GAI helps me think more critically during the writing process.
4. I don’t trust GAI tools to support academic research tasks. (R)

*Affective Dimension*

1. I feel excited about using GAI in my research or teaching.
2. I feel anxious when thinking about integrating GAI into academic work. (R)
3. I find GAI use in academia inspiring and intellectually stimulating.
4. GAI makes me feel uncomfortable about the future of academic integrity. (R)

*Behavioral Dimension*

1. I am likely to incorporate GAI tools in my future academic projects.
2. I avoid using GAI because I worry it will negatively affect my work. (R)
3. I actively seek out new ways to integrate GAI in my academic workflow.
4. I deliberately limit my use of GAI in academic contexts. (R)

**Scale 4 - GAI Academic Misconduct Scale (4 items)**

Instructions: The following items are designed to assess the frequency with which you may have engaged in academic misconduct involving generative artificial intelligence tools (e.g., ChatGPT, DeepSeek, Claude, etc.). Please answer honestly based on your behavior during the most recent academic semester or major academic task.

Response scale:

1 = Never

2 = Rarely

3 = Sometimes

4 = Often

5 = Always

1. I have used generative AI tools to fabricate references (e.g., citing non-existent articles or authors).
2. I have submitted AI-generated artistic works (e.g., music, paintings, or images) as original academic assignments.
3. I have used generative AI to create fake internship or employment verification documents.
4. I have fabricated research data or findings using generative AI tools.
5. I have used AI to answer in unauthorized exams or tests.

Scoring: Add the scores from all five items. Higher total scores indicate a higher frequency of engagement in generative AI-related academic misconduct.

1. Attention check: What’s the topic of this study?

Biology

Artificial intelligence

Law
